# High Iodine Induces the Proliferation of Papillary and Anaplastic Thyroid Cancer Cells *via* AKT/Wee1/CDK1 Axis

**DOI:** 10.3389/fonc.2021.622085

**Published:** 2021-03-16

**Authors:** Chunpeng Lv, Yanhui Gao, Jinyin Yao, Yan Li, Qun Lou, Meichen Zhang, Qiushi Tian, Yanmei Yang, Dianjun Sun

**Affiliations:** ^1^ Center for Endemic Disease Control, Chinese Center for Disease Control and Prevention, Harbin Medical University, Harbin, China; ^2^ Key Laboratory of Etiology and Epidemiology, Education Bureau of Heilongjiang Province & Ministry of Health, Harbin, China

**Keywords:** thyroid cancer, high iodine, proliferation, transcriptome analysis, cyclin-dependent kinase 1 (CDK1), AKT

## Abstract

High iodine can alter the proliferative activity of thyroid cancer cells, but the underlying mechanism has not been fully elucidated. Here, the role of high iodine in the proliferation of thyroid cancer cells was studied. In this study, we demonstrated that high iodine induced the proliferation of BCPAP and 8305C cells *via* accelerating cell cycle progression. The transcriptome analysis showed that there were 295 differentially expressed genes (DEGs) in BCPAP and 8305C cells induced by high iodine, among which CDK1 expression associated with the proliferation of thyroid cancer cells induced by high iodine. Moreover, the western blot analysis revealed that cells exposed to high iodine enhanced the phosphorylation activation of AKT and the expression of phospho-Wee1 (Ser642), while decreasing the expression of phospho-CDK1 (Tyr15). Importantly, the inhibition of AKT phosphorylation revered the expression of CDK1 induced by high iodine and arrested the cell cycle in the G_1_ phase, decreasing the proliferation of thyroid cancer cells induced by high iodine. Taken together, these findings suggested that high iodine induced the proliferation of thyroid cancer cells through AKT-mediated Wee1/CDK1 axis, which provided new insights into the regulation of proliferation of thyroid cancer cells by iodine.

## Introduction

Follicular cell-derived thyroid cancer is the most common endocrine cancer, which can be mainly classified into papillary cancer (PTC), follicular cancer (FTC) and anaplastic cancer (ATC) (1). Among them, PTC is a well-differentiated carcinoma with an excellent prognosis, and accounts for over 80% of the total incidence (2); while ATC is the most aggressive thyroid carcinoma with poor prognosis, only accounting for less than 5% of the incidence, but it accounts for a large proportion of the mortality (2–5). In recent years, thyroid cancer incidence has been obviously increasing worldwide and most of the new cases were attributing to PTC (6–9). Evidences have suggested that increased ability to detect and diagnose or overdiagnosis may be the substantial, but there are also other contributors to this increase (10–12). Some genetic and environmental factors are also suspected to be the risk factors, especially the relationship between iodine excess intake and thyroid cancer (13–16). Thus, it is imminent to explore the implications of high iodine exposure on thyroid cancer.

As an essential trace element for thyroid function, iodine plays an important role in human growth development and metabolism. Either deficient or excessive iodine intakes contribute to the progress of thyroid disease (17). Available evidences suggest that iodine deficiency is a risk factor for thyroid cancer (18, 19), but the effect of iodine excess on thyroid cancer remains controversy (20, 21). Furthermore, studies suggest that iodine intake higher than physiological concentration promotes the growth of thyroid cancer cells (22, 23). However, until now, the mechanism underlying the promotive effect of high iodine on the thyroid cancer cells is not fully elucidated.

As other neoplastic lesions, the development of thyroid cancer results from an imbalance between cellular proliferation and programmed cell death. Ordered regulation of cell cycle is crucial to the survival of a cell and prevention of uncontrolled cell division. Cell cycle checkpoints controlled by numerous proteins are the regulatory mechanism that controls the order and temporal coordination of cell cycle transition (24). There are over 13 diverse CDKs and 25 cyclins in human cells, which can form multiple CDK-cyclin complexes at distinct stages of cell cycle to exert specific effects on cell cycle progression (25). Among them, CDK1 is the key regulator of cell cycle regulation, binds to Cyclin B and forms cyclin B1-CDK1 complex, which is necessary for G_2_/M transformation (26). Overexpression of CDK1 have been found in various type of human cancers, including thyroid cancer (27), ovarian cancer (28), colorectal cancer (29), etc, thus it plays a pivotal role in driving oncogenesis.

Protein kinase B (AKT) is an important signaling molecular in regulating cell survival and proliferation, which promotes cell cycle progression *via* regulating the cell cycle regulators, resulting in cancer development and progression (30). In humans, there are three AKT genes, namely AKT1, AKT2, and AKT3, which is a high degree of amino acid sequence similarity (31). Among them, AKT1 plays a vital role in cell proliferation and survival. There are multiple targets available for AKT1, which stimulates cellular proliferation through downstream substrates with significant implications on cell cycle progression and regulation (32). It is reported that AKT can inhibit Wee1 or Myt1 *via* AKT-dependent phosphorylation, while the phosphorylation of CDK1 at Tyr15 and Thr14 catalyzed by Wee1 and Myt1 results in inhibition of CDK1 and prevents entry into mitosis, thereby promoting the G_2_/M transition (33). Thus, AKT indirectly causes the activation of CDK1 and promotes cell cycle progression at G_2_/M transition. AKT activation has been observed in thyroid cancers, and mediates thyroid cancer development promoted by iodine (34). However, it is unclear whether CDK1 expression regulated by AKT is involved in the proliferation of thyroid cancer cells induced by iodine *via* regulating cell cycle progression.

This study was designed to explore whether AKT-mediated Wee1/CDK1 axis is involved in the proliferation of PTC and ATC cells induced by high iodine, with a new insights that iodine plays the role on cell proliferation in thyroid cancer.

## Materials and Methods

### Chemicals and Solutions

Ly294002 (CAS no.154447-36-6), a specific inhibitor for PI3K/AKT pathway, was purchased from MCE (purity > 99.9%) and dissolved in Dimethyl Sulfoxide (DMSO, Sigma) to yield the working concentrations. It was well-mixed and well-distributed in DMSO prior to and throughout dosing.

### Cell Lines and Cell Culture

The human PTC cell line (BCPAP) and ATC cell line (8305C) were purchased from the cell library of the Chinese Academy of Sciences, which were respectively cultured in RPMI 1640 medium (Thermo Fisher Scientific) or Eagle’s Minimum Essential Medium (Gibco) supplemented with 10% fetal bovine serum (FBS, Gibco). Cells were incubated at 37°C in humidified air containing 5% CO_2_.

### Cell Proliferation Assay

Cells were seeded in 96-well plates (5000 cells/well), cultured over-night and exposed to test agents in complete medium. Cell viability was evaluated by Cell Counting Kit-8 kit (CCK-8, Dojindo) and Ethynyldeoxyuridine assay (EdU, RiboBio) according to the manufacturer’s protocol.

### Cell Cycle Assay

BCPAP and 8305C cells were seeded into 6 well plates (10^6^ cells/well), cultured over-night and exposed to test agents in complete medium. Cell cycle distribution was subsequently analyzed by Cell Cycle and Apoptosis Analysis Kit (Beyotime Biotechnology) according to the manufacturer’s protocol. The cellular DNA content was measured by flow cytometry (Becton Dickinson, BD), and the percentage of cells in each phase of cell cycle was analyzed using the ModFit software.

### Cell Apoptosis Assay

Annexin V-FITC Apoptosis Detection kit (BD, USA) was used to detect cell apoptosis. BCPAP and 8305C (2 × 10^6^ cells/well) were washed twice with cold PBS and resuspended in 1X Binding Buffer. Cells were analyzed with a flow cytometer (BD Accuri C6 Plus, USA). FlowJo V10 was used to calculate the number of viable cells, early apoptotic cells, and late apoptotic cells.

### Quantitative Real-Time RT-PCR

Total RNA was extracted using TRIzol reagent (TaKaRa). cDNA was synthesized with the Prime-Script RT reagent Kit (TaKaRa). RT-qPCR analyses for CDK1, Wee1, AKT1 and β-actin were performed using SYBR Premix ExTaq (TaKaRa). The primers used were listed in **Table S1**. RT-qPCR was performed using the ABI 7500 fast real-time PCR machine (Applied Biosystems). The relative expression of each gene was calculated and normalized using the 2^−ΔΔCt^ method. RT-qPCR was conducted in triplicate for each sample.

### Microarrays Analysis

Total RNA was extracted from three paired samples of BCPAP and 8305C cells treated with or without exposure to high iodine using TRIzol reagent (TaKaRa). cDNA probes were synthesized from the total RNA and subjected to hybridization with a HuGene 2 1 ST array (Affymetrix). Raw intensity data for each experiment were analyzed with the use of Transcriptome Analysis Console (TAC) software (Applied Biosystems).

### Identification Differentially Expressed Genes

The DEGs were screened with |Flod Change| > 1.5 and p-value < 0.05. A Venn diagram was conducted *via* the online tool (https://bioinfogp.cnb.csic.es/tools/venny/) to obtain genes that was regulated by iodine in PTC and ATC cells. Gene Ontology (GO) and Kyoto Encyclopedia of Genes and Genomes (KEGG) pathway analysis were performed with R loading with packages “clusterProfiler,” “enrichplot,” and “ggplot2.” Both p- and false discovery rate (FDR) < 0.05 were considered significantly enriched.

### Western Blot Analysis

The proteins in BCPAP and 8305C cells were extracted using the Total Protein Extraction Kit (Beyotime Biotechnology). The extracted protein (40 μg) were separated by 10% sodium dodecyl sulphate polyacrylamide gel electrophoresis (SDS-PAGE) and then electrophoretically transferred to polyvinylidene fluoride (PVDF) membranes (Millipore). After blocking in 5% non-fat milk for 1 h at room temperature, the membranes were incubated overnight at 4°C with primary antibodies against AKT, p-AKT, Wee1, p-Wee1, Cyclin B and β-actin (1:1000; Cell Signaling Technology), CDK1 (1:1000; Abcam), p -CDK1 (1:1000; R&D), Cyclin A (1:1000; Affinity), caspase 3 (1:250; Affinity) and Cleaved caspase 3 (1:250; Affinity). After incubation with secondary antibodies (1:5000 dilution; CST) for 1 h, TBST was used to wash the protein bands, which were visualized using an enhanced chemiluminescence (ECL) detection reagent (Haigene) and detected using the Tanon imaging system (Tanon).

### Xenograft Tumor Model

Animal experiments were performed in accordance with the National Institutes of Health Guide for the Care and Use of Laboratory Animals, which was approved by the institutional animal care and use committee of Harbin Medical University (hrbmuecdc20180302). For xenograft experiments, twenty BALB/c nude mice of 4–6 weeks old were divided into four groups randomly based on body weights (BCPAP: control, I_6000μg/L_; 8305C: control, I_6000μg/L_; n = 5 for each group). Each mouse was injected with 2 × 10^7^ indicated cells. Tumor volume was calculated by measuring the length and width of the tumor (tumor volume = length×width^2^/2). The measurement was carried out every 3 days. After 21 days in tumor formation, the mice were sacrificed by cervical dislocation and the tumors were removed and weighed for further analysis, and the blood sampled isolated from by eyeball extirpating.

### Determination of Iodine Concentration in Serum

Iodine content in serum was detected by As3^+^-Ce4^+^ catalytic spectrophotometry based on the nationally standardized method in China (WS/T572-2017). Briefly, 0.5 ml perchloric acid and 0.6 ml sodium chlorate, respectively, was added to 0.20 ml blood samples, vortex-mixed, digested at 130°C for 2 h, 0.6 ml ammonium ceric sulfate were added after adding 3 ml arsenic acid and iodine concentration in diluted blood and standard samples with different concentrations were tested by a double-beam UV-Vis Spectrophotometer (Beijing Persee General Instruments Co. Ltd).

### Immunohistochemical Analysis

All specimens were fixed in 4% paraformaldehyde, embedded in paraffin and then sectioned at a thickness of 4μm. Briefly, the sections were treated with 3.0% H2O2 for 10 min to block the endogenous peroxidase activity. Each section was subjected to microwave antigen retrieval with citrate buffer (0.01%, pH 6.0) and blocked with 5% bovine serum albumin for 30 min. Then, the sections were incubated in a humidity chamber with the following antibodies overnight at 4°C: PCNA (1:400; #13110; CST, USA), CDK1 (1:100; #133327; Abcam), p-CDK1 (1:100; R&D), Wee1 (1:150; #203236; Abcam), p-Wee1 (1:100; #203236; Abcam), AKT (1:100; #4691; CST), p-AKT (1:50; #AF0016; Affinity), caspase 3 (1:50; #AF6311; Affinity) or Cleaved caspase 3 (1:50; #AF7022; Affinity). Next, sections were incubated with a one-step polymer detection for mouse/rabbit antibodies (ZSGB Biotechnology) in a humidity chamber for 20 min at room temperature, incubated (for color development) in a DAB staining kit (ZSGB Biotechnology) and finally counterstained with hematoxylin for 1 min. Staining scores were observed in terms of staining intensity and percentage of positively stained cells. Staining intensity was divided into four grades: 0 (no staining), 1 (weak staining), 2 (intermediate staining), or 3 (strong staining). The staining percentages were divided into the following grades: 0 (<5% positive), 1 (<25% positive), 2 (25%–50% positive), 3 (51%–75% positive), and 4 (>75% positive) (35). Independent fields (×400) were randomly selected to obtain an average score.

### Statistical Analysis

Statistical analysis was carried out using SPSS 23.0 (IBM). Data were expressed as mean ± SD and were analyzed with independent samples t-test or one-way ANOVA. The *post hoc* analysis was carried out to compare the significance between groups by Dunnett T_3_ test or LSD test. Differences in tumor volume among iodine treatments and control groups were compared using the repeated measure of ANOVA, and differences in tumor weight and iodine concentration in serum between iodine treatments and control groups were compared using Mann-Whitney U test or independent samples t-test. P < 0.05 was considered statistically significant.

## Results

### High Iodine Promotes the Proliferation of BCPAP and 8305C Cells *via* Regulating Cell Cycle Progression

**Figure 1:**
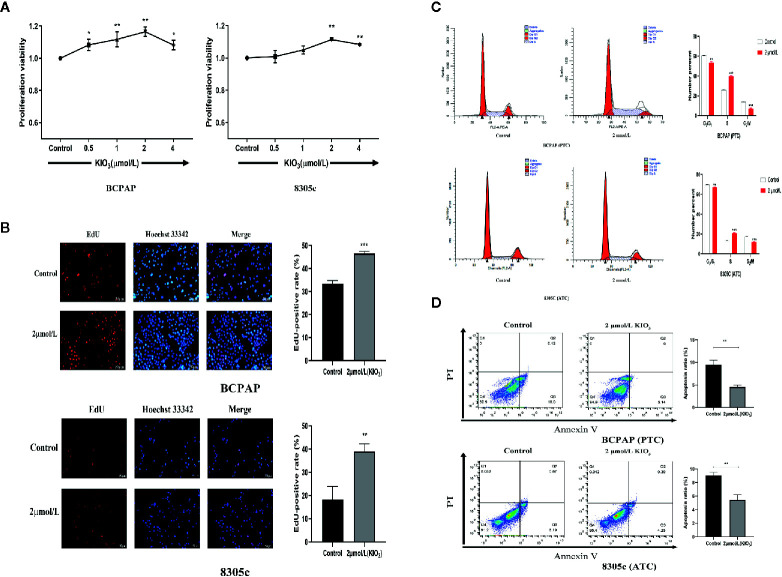
High iodine promoted the proliferation of thyroid cancer cells. **(A)** BCPAP and 8305C cells were treated with gradient concentrations of high iodine for 72 h. Cell proliferation was evaluated by CCK-8 assays. **(B)** BCPAP and 8305C cells were treated with 2 μmol/L KIO_3_ treatment for 72 h. Cell viability was evaluated by EdU assays. **(C)** Flow cytometry analysis of G_1_, S, and G_2_/M phases in BCPAP and 8305C cells that were treated with or without high iodine. **(D)** Apoptosis rate of BCPAP and 8305C cells induced by high iodine for 72 h were detected by flow cytometry. *P < 0.05, **P < 0.01, ***P < 0.001.

CCK-8 assays were used to identify the effect of high iodine on the proliferation of BCPAP and 8305C cells. As shown in **Figure 1A**, after treatments with 2 μmol/L KIO_3_ for 72 h, the viability of BCPAP and 8305C cells increased up to 18% and 11% (*P* < 0.05), respectively. Therefore, treatment with 2 μmol/L KIO_3_ for 72 h was used as the dose and time of KIO_3_ treatment in the following experiments.

The effect of high iodine on DNA synthesis in BCPAP and 8305C cells was examined by EdU assay, a sensitive and robust method to evaluate cell proliferation. As shown in **Figure 1B**, compared with the control groups, treatment with 2 μmol/L KIO_3_ for 72 h increased the EdU-positive rate of BCPAP and 8305C cells by 27.5% and 53.4%, respectively. These results indicated that exposure to high iodine promoted the proliferation of BCPAP and 8305C cells.

Next, flow cytometry was used to analyze the effect of high iodine on the cell cycle in BCPAP and 8305C cells. As shown in **Figure 1C**, compared with the control groups, treatment with 2 μmol/L KIO_3_ for 72 h significantly decreased the proportion of G_1_ and G_2_ phase cells but increased the proportion of S phase cells in both BCPAP and 8305C cells (*P* < 0.05). These results indicated that high iodine may regulate cell cycle in BCPAP and 8305C cells.

In addition, we evaluated apoptosis using the Annexin-V-FITC/PI method. Flow cytometry results showed that the apoptotic rate of BCPAP and 8305C cells decreased with exposure to 2 μmol/L KIO_3_ for 72 h (**Figure 1D**). The average percentage of apoptotic cells in the high-dose group was significantly lower than that in the control group (for BCPAP cells: 9.489% *vs.* 4.56%; for 8305C cells: 9.02% *vs.* 5.43, *P* < 0.01). These results indicated that high iodine may promote the proliferation of BCPAP and 8305C cells and inhibit apoptosis.

### Differential Expression Genes and Enrichment Analysis

**Figure 2:**
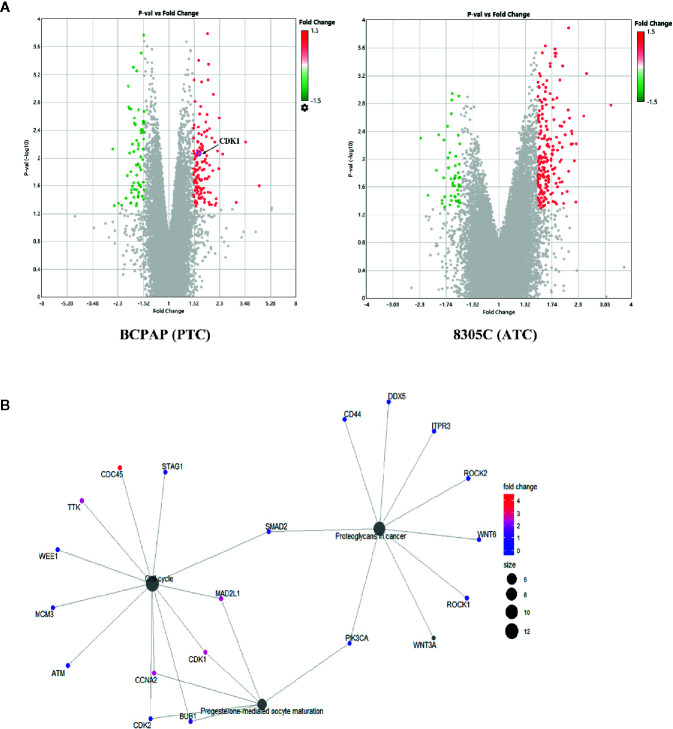
Identification of genes related to high iodine. **(A)** Volcano plot of DEGs in BCPAP and 8305C cells were treated with or without high iodine. Red/green symbols classify the upregulated/downregulated genes according to the criteria: |Fold Change| >1.5 and P-value < 0.05. **(B)** An interactive network between genes and pathways based on DEGs from KEGG enrichment analysis.

To explore the molecular mechanisms that high iodine induced the proliferation of thyroid cancer cells, gene microarray were used to conduct a comprehensive analysis. As shown in the volcano plots, 153 and 149 DEGs were identified which regulated by high iodine in the BCPAP and 8305C cells compared to control groups (**Figure 2A**). As shown in the Venn diagram, a total of 295 DEGs were significantly regulated in BCPAP and 8305C cells with exposure to high iodine, in which the seven genes were identified (DIAPH3, KIF20B, LINC00472, LOC105374715, PHIP, SGOL1, and ZNF581) in both BCPAP and 8305C cells (**Figure S1**). These results indicated these genes played a critical role in the proliferation of thyroid cancer cells induced by high iodine.

In present study, the GO/KEGG enrichment analysis was applied based on DEGs to classify significantly enriched pathways. For GO analysis, these influences of DEGs on cellular GO enrichment terms are shown in **Figure S2A**, including cellular process, cellular anatomical entity, membrane-bounded organelle, intracellular organelle, cytoplasm, etc. For KEGG analysis, 63 pathways regulated by iodine were significantly enriched, including cell cycle, PI3K/AKT pathway, transcriptional misregulation in cancer, etc (**Figure S2B**). Importantly, a network of interactions between genes and pathways based on 295 DEGs was identified (**Figure 2B**), suggesting that cell cycle pathway is closely related to CDK1.

In addition, protein-protein interaction (PPI) network were constructed *via* the STRING database and used Cytoscape software to identify significant genes that regulated by high iodine. The interactions among 295 genes are shown in **Figure S3**, and the bar plots were represented for the top 10 genes ranked by the number of nodes software. Finally, CDK1, as the most significant gene, was identified from the above analysis.

### High Iodine Significantly Increases AKT/Wee1/CDK1 Expression in the Thyroid Cancer Cells

**Figure 3:**
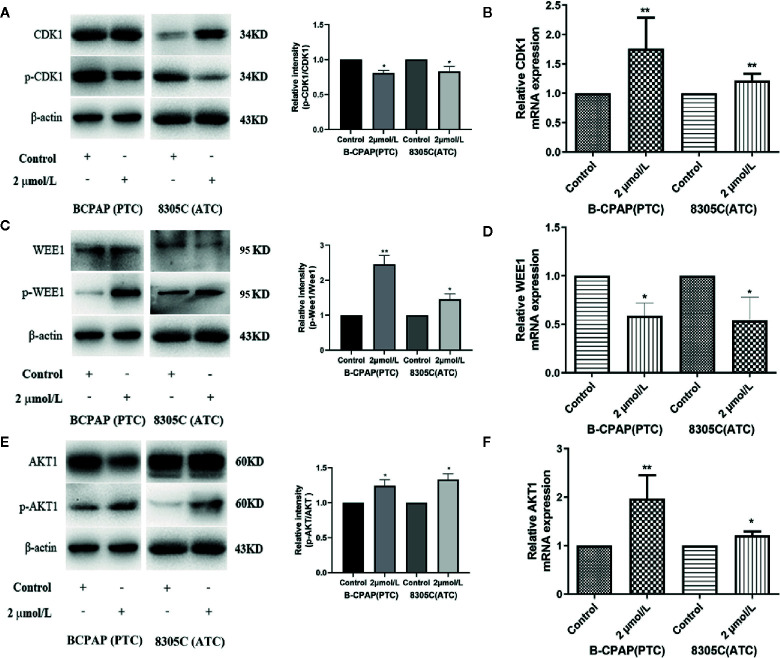
High iodine increased AKT/Wee1/CDK1 expression in BCPAP and 8305C cells. Data were presented as means ± SD. **(A)** Western blot of CDK1 and p-CDK1 in BCPAP and 8305C cells treated with or without 2 μmol/L KIO_3_ for 72 h. **(B)** RT-qPCR was used to detect the expression of CDK1 in BCPAP and 8305C cells treated with or without 2 μmol/L KIO_3_ for 72 h. **(C)** Western blot of Wee1 and p-Wee1 in BCPAP and 8305C cells treated with or without 2 μmol/L KIO_3_ for 72 h. **(D)** RT-qPCR was used to detect the expression of Wee1 in BCPAP and 8305C cells treated with or without 2 μmol/L KIO_3_ for 72 h. **(E)** Western blot of AKT and p-AKT in BCPAP and 8305C cells treated with or without 2 μmol/L KIO_3_ for 72 h. **(F)** RT-qPCR was used to detect the expression of AKT1 in BCPAP and 8305C cells treated with or without 2 μmol/L KIO_3_ for 72 h. *P < 0.05, **P < 0.01.

As mentioned above, CDK1, as a significant hub gene in PPI network, was identified, the protein and mRNA expression of that was detected to determine whether high iodine stimulated their expression in BCPAP and 8305C cells. Shown in **Figures 3A, B**, the expressions of CDK1 were both significantly increased in mRNA (*P* < 0.01) and protein level (*P* < 0.05) in BCPAP and 8305C cells treated by high iodine compared with those in the control.

Due to the interaction among CDK1, Wee1 and AKT1 as well as their crucial role in the cell cycle progression, the expression of Wee1 and AKT1 were detected in mRNA and protein level. As shown in **Figures 3C, E**, western blot analyses indicated that treatment with 2 μmol/L KIO_3_ for 72 h significantly increased the levels of phospho-AKT (Ser473) and phospho-Wee1 (Ser642) in both cell lines, whereas the levels of AKT and Wee1 were unchanged, thereby elevating the ratio of p-AKT/AKT (*P* < 0.05) and p-Wee1/Wee1 (*P* < 0.01) in BCPAP and 8305C cells. Similarly, shown in **Figures 3D, F**, the expression of AKT1 in mRNA level (*P* < 0.05) was significantly increased in BCPAP and 8305C cells treated with high iodine compared with those in the control groups, while the expression of Wee1 in mRNA level (*P* < 0.05) decreased in an opposite manner. These results indicated that high iodine significantly increased AKT/Wee1/CDK1 expression in thyroid cancer cells. Since CDK1 is partnered by cyclin A and cyclin B, we examined the expression of Cyclin A and Cyclin B protein in BCPAP and 8305C cells treated with high iodine. The results showed that the expression of Cyclin A and Cyclin B protein was no significant difference between these groups (**Figure S4**). In addition, high iodine can inhibit the apoptosis of BCPAP and 8305C cells. And, western blot results showed that the ratio of cleaved-caspase3/caspase3 significantly decreased in BCPAP and 8305C cells treated with high iodine compared with the control group (*P <*0.01, **Figure S4**).

### High Iodine Promotes the Cell Proliferation *via* AKT/Wee1/CDK1 Axis

**Figure 4:**
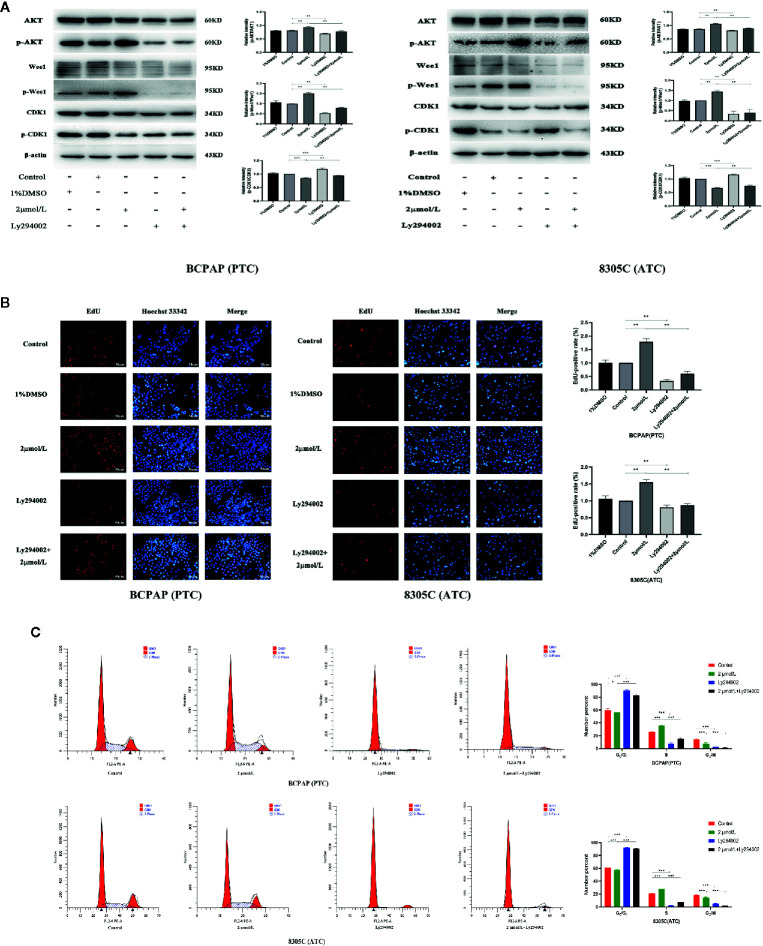
High iodine induced the proliferation of BCPAP and 8305C cells *via* activating AKT-mediated Wee1/CDK1 axis. Data were presented as means ± SD. **(A)** Ly294002 inhibited the expression levels of p-CDK1/CDK1, p-Wee1/Wee1 and p-AKT/AKT in BCPAP and 8305C cells induced by high iodine. β-actin was used as a loading control. **(B)** Ly294002 inhibited the proliferation of BCPAP and 8305C cells induced by high iodine. Cell viability was measured by the EdU assay, and DMSO was used as a control. **(C)** The proportion of cells in each phase of the cell cycle was evaluated by flow cytometry after 48 h of treatment with Ly294002. *P < 0.05, **P < 0.01, ***P < 0.001.

To explore whether AKT-mediated Wee1/CDK1 is involved in the proliferation of BCPAP and 8305C cells induced by high iodine, we abrogated the induction of AKT activation induced by iodine in these cell lines *via* treatment with Ly294002, an inhibitor of AKT. As a result, treatment with Ly294002 alone decreased the ratio of p-Wee1/Wee1, but increased the ratio of p-CDK1/CDK1, compared with control groups. Besides, pretreatment with Ly294002 in iodine-treated cells decreased the ratio of p-Wee1/Wee1, but increased the ratio of p-CDK1/CDK1, compared with high iodine-treated groups (**Figure 4A**). However, the expression of Cyclin A and Cyclin B in iodine combined with inhibitor-treated group was not statistically significant, compared with high iodine-treated group (**Figure S5**). Furthermore, pretreatment with Ly294002 reduced the EdU-positive cells induced by high iodine in these cell lines (**Figure 4B**), and arrested the cell cycle in the G_1_ phase, which reduced the proportion of cells in the S/G_2_ phase (**Figure 4C**). These results indicated that high iodine can accelerate the cell cycle progression and induce the proliferation of BCPAP and 8305C cells by activating AKT/Wee1/CDK1 axis.

In addition, compared with control groups, treatment with Ly294002 alone increased the ratio of cleaved caspase 3/caspase 3. And, compared with high iodine groups, there was a similar increase in the iodine combined with inhibitor group (*P* < 0.001, **Figure S5**). Flow cytometry results showed that the apoptotic rate of BCPAP and 8305C cells in iodine combined with inhibitor-treated group was significantly higher than that in the high iodine group (*P* < 0.05, **Figure S6**). These results confirmed that high iodine can inhibit the apoptosis of BCPAP and 8305C cells through activating AKT.

### Effects of High Iodine on Tumor Growth in Xenograft Tumor Models of PTC and ATC Cells

**Figure 5:**
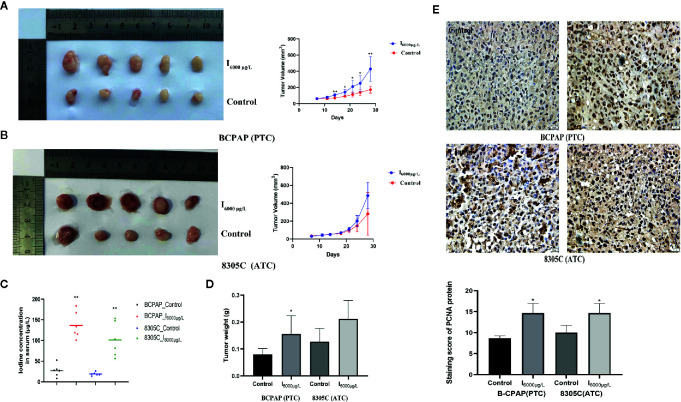
High iodine promoted tumor growth in xenograft tumor models of PTC and ATC cells. All data were presented as means ± SD. **(A, B)** BCPAP and 8305C cells were subcutaneous injected into nude mice under exposure to high iodine. Relative tumor volumes of different groups, monitored every 3 days during treated with or without high iodine. **(C)** Tumor weights were measured at the end of the day 21. **(D)** Iodine concentration in serum of nude mice among control and iodine treatment groups. **(E)** Representative IHC staining images showed staining score of PCNA protein in xenograft tumor tissues. *P < 0.05, **P < 0.01.

To study the effect of high iodine on the proliferation of thyroid cancer cells, we constructed xenograft models of PTC and ATC cells. As shown in **Figures 5A–C**, the tumor volume and weight in PTC cells differed significantly across excessive iodine groups compared to control group (*P* < 0.05), with no significant difference in ATC cells. However, serum iodine was increased in mice wearing both cells of I_6000_ μg/L groups compared to control group (*P* < 0.05, **Figure 5D**). Also, PCNA expression significantly increased in I_6000_ μg/L groups compared to control groups (*P* < 0.05, **Figure 5E**). These indicated that high iodine promoted tumor growth in xenograft tumor models of PTC and ATC cells. In addition, IHC analysis showed that compared to control groups, the ratio of cleaved caspase 3/caspase 3 (*P* < 0.05, **Figure S7**) in xenograft tumor models were significantly decreased in I_6000_ μg/L groups.

### Effects of High Water Iodine on Expression of AKT/Wee1/CDK1 in Xenograft Tumor Models of PTC and ATC Cells

**Figure 6:**
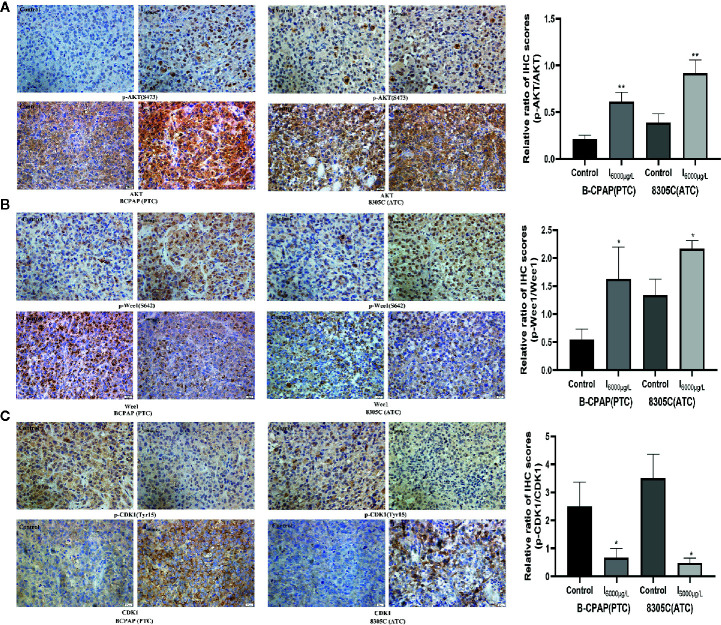
Effects of high water iodine on expression of AKT/Wee1/CDK1 in xenograft tumor models of PTC and ATC cells. Data were presented as means ± SD. Representative photomicrographs of IHC in the xenograft tumors (400 ×). **(A)** Staining score of p-AKT and AKT protein in xenograft tumor tissues among control and iodine treatment groups. **(B)** Staining score of p-Wee1 and Wee1 protein in xenograft tumor tissues among control and iodine treatment groups. **(C)** Staining score of p-CDK1 and CDK1 protein in xenograft tumor tissues among control and iodine treatment groups. *P < 0.05, **P < 0.01.

Since previous experiments found that high iodine can induce the proliferation of BCPAP and 8305C cells by activating AKT/Wee1/CDK1 axis, we discussed whether AKT/Wee1/CDK1 was involved in the tumor growth of xenograft tumor models of PTC and ATC cells induced by high iodine. IHC analysis showed the ratio of p-AKT/AKT (*P* < 0.01, **Figure 6A**) and p-Wee1/Wee1 (*P* < 0.05, **Figure 6B**) in xenograft tumor models were significantly increased in I_6000_ μg/L groups compared to control groups, whereas the ratio of p-CDK1/CDK1 was significantly decreased in the I_6000_ μg/L group compared to control groups (*P* < 0.05, **Figure 6C**). These results suggested that high iodine significantly increased AKT/Wee1/CDK1 expression in xenograft tumor models of PTC and ATC cells.

## Discussion

Thyroid cancer occurrence is a process involving multi-step, multi-factor and multi-gene alterations, in which out-of-control cell proliferation are considered important mechanisms associated with abnormal cell cycle regulation (36). In the current study, we highlighted that exposure to high iodine could promote the proliferation of thyroid cancer cells. Further studies uncovered that high iodine exerted its roles by activating AKT/Wee1/CDK1 axis and accelerating cell cycle progression in thyroid cancer. The above findings provide strong evidences to support the fact that high iodine plays vital roles in the development of thyroid cancer.

Iodine is crucial to thyroid health since this trace element is essential for thyroid hormone synthesis. Both deficient and excessive iodine intakes can cause thyroid disease, so iodine intake-associated health disorders has became the major public health issues (2, 37). Among them, the association between iodine intake and thyroid cancer, especially the effect of high iodine on thyroid cancer incidence, has aroused extensive attention. Several studies reported an increased risk of thyroid cancer in populations living in iodine excessive areas, such as Iceland and Hawaii, but volcanic activity are likely to be responsible for the high incidence of thyroid cancer in these areas rather than iodine excess (20, 38). Another study by Truong et al., performed among native and European residents in New Caledonia, failed to find the relationship between high dietary iodine intake and risk of thyroid cancer (39). Therefore, the association between excess iodine intake and thyroid cancer incidence remain controversy. Previous experiments *in vitro* have demonstrated iodine at a certain concentration range promoted the proliferation of thyroid cancer cells (22, 23). However, the underlying mechanism by which iodine affects thyroid cancer cells is not fully elucidated.

A previous study showed that the normal physiological concentration of iodine in human thyroid is 10^-6^ to 10^-5^ mM, and incubation with iodine at concentration of 10^-3^ mM for 72 h could promote the proliferation of thyroid cancer cells (W3 and FTC-133) (22). Also, we demonstrated that treatment with 2 μmol/L KIO3 for 72 h increased the proliferation of thyroid cancer cells (BCPAP and 8305C). Thus, at the cellular level, treatment with 2 μmol/L KIO3 for 72 h was used as the test dose and time in the following experiments. In addition, previous studies have shown that 6000 μg/L I- is a reasonable and representative dose for human exposure (40). Hence, in xenograft tumor models, an iodine dosage of 6000 μg/L I- was selected to explore the effects of iodine intake on tumor growth.

In this study, results showed that treatment with 2 μmol/L KIO_3_ for 72 h promoted the proliferation of thyroid cancer cells and the proportion of S phase cells, but significantly decreased the proportion of G_1_ and G_2_ phase cells, suggesting that high iodine might promote the proliferation of thyroid cancer cells *via* regulating cell cycle progression. In addition, high iodine had a weak inhibitory effect on apoptosis of thyroid cancer cells. To explore the underlying mechanism, the transcriptional profile was analyzed in BCPAP and 8305C cell exposed to high iodine. The results showed there were 153 and 149 genes in the PTC and ATC cells regulated by high iodine, separately, and the seven genes were identified by overlapping these genes, namely DIAPH3, KIF20B, LINC00472, LOC105374715, PHIP, SGOL1, and ZNF581. But the biological function of seven genes was not reported to be related with the cell cycle regulation and cell apoptosis. Given that, we analyzed the 295 DEGs in the union of BCPAP and 8305C regulated by high iodine. KEGG enrichment analysis of iodine-related genes showed that these genes are mainly correlated with various signaling pathways, including cell cycle, PI3K/AKT signaling, among which apoptosis pathway was not found. As a result, this study mainly focused on the effect of high iodine on the proliferation of thyroid cancer cells *via* regulating cell cycle progression. Importantly, the interaction between genes and pathway showed DEGs were differentially regulated and affected the pathways of the cell cycle, proteoglycans in cancer and progesterone-mediated oocyte maturation. Among them, CDK1 was associated with cell cycle pathways and was involved in proliferation of thyroid cancer cells induced by high iodine.

CDK1, a cylcin-dependent kinase1, is one of the most pleiotropic cell cycle regulators, which plays a vital role in the physiological processes, including cell proliferation, chromosome condensation and chromosome segregation in mitosis (27). It can regulate cell cycle progression *via* interacting with interphase cyclins, such as cyclin A, B, D1, and E (41). In this study, the expression of Cyclin A and Cyclin B had no change in BCPAP and 8305C cells under high iodine treatment, but the phosphorylation of CDK1 kinase at Tyr15 was inhibited by high iodine, thereby increasing CDK1 activity. These results suggested that increased CDK1 activity plays an important role in the proliferation of thyroid cancer cells induced by high iodine. Furthermore, research described that CDK1 was inactivated by Wee1 kinases through catalyzing the inhibitory phosphorylation of CDK1 kinase at Tyr15, whereas Wee1 can be negatively regulated by AKT in a phosphorylation dependent manner (42). Also, in this study, the expression of p-Wee1 (Ser642) and p-AKT (Ser473) were significantly increased in BCPAP and 8305C cells under exposure to high iodine. Therefore, we inferred that AKT/Wee1/CDK1 axis might be activated under exposure to high iodine, thereby promoting the proliferation of developed thyroid cancer cells.

AKT, also known as protein kinase B, is an important intermediate in the process of cell signaling, and can promote the occurrence and development of tumors by regulating biological processes, such as cell proliferation, migration and differentiation (43, 44). In this study, we found that AKT activation might be involved in the proliferation of BCPAP and 8305C cells induced by high iodine, which was similar with previous studies (22, 34). More importantly, the inhibition of AKT activation by Ly294002 not only reduced the induction of high iodine on the expression of CDK1 but reversed the proliferation activity of BCPAP and 8305C cells *via* arresting the cell cycle in the G_1_ phase. These results suggested high iodine induced the proliferation of BCPAP and 8305C cells through activating AKT-mediated Wee1/CDK1 axis. Similarly, in nude mice, we confirmed that AKT/Wee1/CDK1 axis was also involved in tumor growth during exposure to high iodine.

This study shows that high iodine has a proliferative effect on papillary and anaplastic thyroid cancer cells *via* accelerating cell cycle progression. Furthermore, activating AKT/Wee1/CDK1 axis was involved in the proliferation of papillary and anaplastic thyroid cancer cells induced by high iodine. In summary, this study might be helpful for understanding of the complex mechanism underlying high iodine-induced proliferation of thyroid cancer cells, and provide new insight into the relationship between high iodine and thyroid cancer. In addition, this study also had limitations about the classification of tumors to different subtypes as with all research. Although we provided papillary and anaplastic thyroid cancer to explore the underlying mechanism that high iodine promoted the proliferative activity of thyroid cancer cells, it may not be very comprehensive understanding for follicular thyroid cancer subtype. This should be further validated through a series of experiments.

## Data Availability Statement 

The datasets used and analyzed during the current study are available from the corresponding author on reasonable request. Requests to access these datasets should be directed to DS, hrbmusdj@163.com.

## Ethics Statement

Animal experiments were performed in accordance with the National Institutes of Health Guide for the Care and Use of Laboratory Animals, which was approved by the institutional animal care and use committee of Harbin Medical University.

## Author Contributions

Performed the experiments and analyzed the data, MZ, QT, CL, JY, YL, and QL. Writing – original & draft, CL. Writing – review & editing, YG, YY, and DS. Funding acquisition, DS. All authors contributed to the article and approved the submitted version.

## Funding

This work was supported by the Key Programme of National Natural Science Foundation of China (Grant No.81830098).

## Conflict of Interest

The authors declare that the research was conducted in the absence of any commercial or financial relationships that could be construed as a potential conflict of interest.

## Supplementary Material

Supplementary Figure 1 The Venn calculation result obtained genes that involved in the proliferation of BCPAP and 8305C cells induced by high iodine. The 2 datasets showed an overlap of 295 genes.

Supplementary Figure 2 Enrichment analysis for 295 DEGs related to iodine. Terms with *p* and *q* < 0.05 were believed to be enriched significantly. The top ten pathways were displayed. **(A)** GO analysis for 295 DEGs related to iodine. **(B)** KEGG enrichment analysis for 295 DEGs related to iodine

Supplementary Figure 3 Protein–protein interaction network based on DEGs related to iodine. **(A)** Interaction network constructed with the nodes with interaction confidence value > 0.40. **(B)** The top 10 genes ordered by degree method. **(C)** The top 10 genes ordered by the number of nodes

Supplementary Figure 4 Effect of high iodine on the expression of Cyclin A, Cyclin B, caspase 3 and cleaved caspase 3 in BCPAP and 8305C cells. Data were presented as means ± SD. β-actin was used as a loading control. **P* < 0.05, ***P* < 0.01, ****P* < 0.001.

Supplementary Figure 5 Effects of high iodine on expression of Cyclin A, Cyclin B, caspase 3 and cleaved caspase 3 in BCPAP and 8305C cells under treatment with Ly294002. Data were presented as means ± SD. ’-actin was used as a loading control. **P* < 0.05, ***P* < 0.01, ****P* < 0.001.

Supplementary Figure 6 Apoptosis rate of BCPAP and 8305C cells under treatment with Ly294002 were detected by flow cytometry. *P < 0.05, **P < 0.01, ***P < 0.001.

Supplementary Figure 7 Effects of high water iodine on the expression of caspase 3 and cleaved caspase 3 in xenograft tumor models of PTC and ATC cells. Data were presented as means ± SD. Representative photomicrographs of IHC in the xenograft tumors (400 ×). **P* < 0.05, ***P* < 0.01.

## References

[1] Xing M. Molecular pathogenesis and mechanisms of thyroid cancer. Nat Rev Cancer (2013) 13(3):184–99. 10.1038/nrc3431 PMC3791171 23429735

[2] Zimmermann MB Boelaert K. Iodine deficiency and thyroid disorders. Lancet Diabetes Endocrinol (2015) 3(4):286–95. 10.1016/S2213-8587(14)70225-6 25591468

[3] Cornett WR Sharma AK Day TA Richardson MS Hoda RS van Heerden JA. Anaplastic thyroid carcinoma: an overview. Curr Oncol Rep (2007) 9(2):152–8. 10.1007/s11912-007-0014-3 17288883

[4] Keutgen XM Sadowski SM Kebebew E. Management of anaplastic thyroid cancer. Gland Surg (2015) 4(1):44. 10.3978/j.issn.2227-684X.2014.12.02 25713779 PMC4321056

[5] Molinaro E Romei C Biagini A Sabini E Agate L Mazzeo S. Anaplastic thyroid carcinoma: from clinicopathology to genetics and advanced therapies. Nat Rev Endocrinol (2017) 1311(11):644–60. 10.1038/nrendo.2017.76 28707679

[6] Pellegriti G Frasca F Regalbuto C Squatrito S Vigneri R. Worldwide increasing incidence of thyroid cancer: update on epidemiology and risk factors. J Cancer Epidemiol (2013) 2013:965212. 10.1155/2013/965212 23737785 PMC3664492

[7] Lim H Devesa SS Sosa JA Check D Kitahara CM. Trends in Thyroid Cancer Incidence and Mortality in the United States, 1974-2013. JAMA (2017) 317(13):1338–48. 10.1001/jama.2017.2719 PMC8216772 28362912

[8] La Vecchia C Malvezzi M Bosetti C Garavello W Bertuccio P Levi F. Thyroid cancer mortality and incidence: a global overview. Int J Cancer (2015) 136(9):2187–95. 10.1002/ijc.29251 25284703

[9] Chen W Zheng R Baade PD Zhang S Zeng H Bray F. Cancer statistics in China, 2015. CA Cancer J Clin (2016) 66(2):115–32. 10.3322/caac.21338 26808342

[10] Udelsman R Zhang Y. The epidemic of thyroid cancer in the United States: the role of endocrinologists and ultrasounds. Thyroid (2014) 24(3):472–9. 10.1089/thy.2013.0257 PMC3949447 23937391

[11] Cabanillas ME McFadden DG Durante C. Thyroid cancer. Lancet (2016) 388(10061):2783–95. 10.1016/S0140-6736(16)30172-6 27240885

[12] Vaccarella S Franceschi S Bray F Wild CP Plummer M Dal Maso L. Worldwide thyroid-cancer epidemic? The increasing impact of overdiagnosis. N Engl J Med (2016) 375(7):614–7. 10.1056/NEJMp1604412 27532827

[13] Zimmermann MB Galetti V. Iodine intake as a risk factor for thyroid cancer: a comprehensive review of animal and human studies. J Thyroid Res (2015) (8):8. 10.1186/s13044-015-0020-8 PMC4490680 26146517

[14] Renehan A Tyson M Egger M Heller R Zwahlen M. Body-mass index and incidence of cancer: a systematic review and meta-analysis of prospective observational studies. Lancet (London England) (2008) 371(9612):569–78. 10.1016/s0140-6736(08)60269-x 18280327

[15] Ito Y Nikiforov YE Schlumberger M Vigneri R. Increasing incidence of thyroid cancer: controversies explored. Nat Rev Endocrinol (2013) 9(3):178–84. 10.1038/nrendo.2012.257 23358352

[16] Zhao H Li H Huang T. High iodine intake and central lymph node metastasis risk of papillary thyroid cancer. J Trace Elements Med Biol (2019) 53(5):16–21. 10.1016/j.jtemb.2019.01.015 30910201

[17] Laurberg P Pedersen IB Knudsen N Ovesen L Andersen S. Environmental iodine intake affects the type of nonmalignant thyroid disease. Thyroid (2001) 11(5):457. 10.1089/105072501300176417 11396704

[18] Hill RN Erdreich LS Paynter OE Roberts PA Rosenthal SL Wilkinson CF. Thyroid follicular cell carcinogenesis. Toxicol Sci (1989) 12(4):629–97. 10.1093/toxsci/12.4.629 2663577

[19] Galanti MR Sparén P Karlsson A Grimelius L Ekbom A. Is residence in areas of endemic goiter a risk factor for thyroid cancer? Int J Cancer (1995) 61(5):615–21. 10.1002/ijc.2910610506 7768633

[20] Feldt-Rasmussen U. Iodine and cancer. Thyroid (2001) 11(5):483–6. 10.1089/105072501300176435 11396706

[21] Lv C Yang Y Jiang L Gao L Rong S Darko GM. Association between chronic exposure to different water iodine and thyroid cancer: a retrospective study from 1995 to 2014. Sci Total Environ (2017) 609:735–41. 10.1016/j.scitotenv.2017.07.101 28763670

[22] Xiang J Wang X Wang Z Wu Y Li D Shen Q. Effect of different iodine concentrations on well-differentiated thyroid cancer cell behavior and its inner mechanism. Cell Biochem Biophys (2015) 71(1):299–305. 10.1007/s12013-014-0198-8 25120024

[23] Liu Y Li H Zhang J Gao X. Potassium Iodate Differently Regulates the Proliferation, Migration, and Invasion of Human Thyroid Cancer Cells via Modulating miR-146a. Cancer Invest (2017) 35(2):122–8. 10.1080/07357907.2016.1261883 28103112

[24] Elledge SJ. Cell cycle checkpoints: preventing an identity crisis. Science (1996) 274(5293):1664–72. 10.1126/science.274.5293.1664 8939848

[25] Liao Y Feng Y Shen J Hornicek FJ Duan Z. The roles and therapeutic potential of cyclin-dependent kinases (CDKs) in sarcoma. Cancer Metastasis Rev (2016) 35(2):151–63. 10.1007/s10555-015-9601-1 26669603

[26] Mir S De Witt Hamer P Krawczyk P Balaj L Claes A Niers J. In silico analysis of kinase expression identifies WEE1 as a gatekeeper against mitotic catastrophe in glioblastoma. Cancer Cell (2010) 18(3):244–57. 10.1016/j.ccr.2010.08.011 PMC3115571 20832752

[27] Zheng H-P Huang Z-G He R-Q Lu H-P Chen G. Integrated assessment of CDK1 upregulation in thyroid cancer. Am J Trans Res (2019) 11(12):7233–54. PMC6943461 31934275

[28] Wang L-L Sun K-X Wu D-D Xiu Y-L Chen X Chen S. DLEU1 contributes to ovarian carcinoma tumourigenesis and development by interacting with miR-490-3p and altering CDK1 expression. J Cell Mol Med (2017) 21(11):3055–65. 10.1111/jcmm.13217 PMC5661118 28598010

[29] Sung W-W Lin Y-M Wu P-R Yen H-H Lai H-W Su T-C. High nuclear/cytoplasmic ratio of Cdk1 expression predicts poor prognosis in colorectal cancer patients. BMC Cancer (2014) 14(1):951. 10.1186/1471-2407-14-951 25511643 PMC4302138

[30] Veronique N Patra KC Nissim H. Selective eradication of cancer displaying hyperactive Akt by exploiting the metabolic consequences of Akt activation. Elife Sci (2018) 7:e32213–. 10.7554/eLife.32213 PMC5980228 29687779

[31] Vanhaesebroeck B Alessi DR. The PI3K–PDK1 connection: more than just a road to PKB. Biochem J (2000) 346(3):561–76. 10.1042/bj3460561 PMC1220886 10698680

[32] Duggal S Jailkhani N Midha MK Agrawal N Rao KVS Kumar A. Defining the Akt1 interactome and its role in regulating the cell cycle. Sci Rep (2018) 8(1):1303. 10.1038/s41598-018-19689-0 29358593 PMC5778034

[33] Lewis CW Bukhari AB Xiao EJ Choi WS Smith JD Homola E. Upregulation of Myt1 Promotes Acquired Resistance of Cancer Cells to Wee1 Inhibition. Cancer Res (2019) 79(23):5971–85. 10.1158/0008-5472.can-19-1961 31594837

[34] Yang X Sun J Han J Sun L Wang H Zhang D. Iodine promotes thyroid cancer development via SPANXA1 through the PI3K/AKT signalling pathway. Oncol Lett (2019) 18(1):637–44. 10.3892/ol.2019.10391 PMC6546993 31289536

[35] Xu Q Liu X Liu Z Zhou Z Tu K. MicroRNA-1296 inhibits metastasis and epithelial-mesenchymal transition of hepatocellular carcinoma by targeting SRPK1-mediated PI3K/AKT pathway. Mol Cancer (2017) 16(1):103. 10.1186/s12943-017-0675-y 28606154 PMC5469159

[36] Marlowe JL Puga A. Aryl hydrocarbon receptor, cell cycle regulation, toxicity, and tumorigenesis. J Cell Biochem (2005) 96(6):1174–84. 10.1002/jcb.20656 16211578

[37] Teng W Shan Z Teng X Guan H Li Y Teng D. Effect of iodine intake on thyroid diseases in China. New Engl J Med (2006) 354(26):2783–93. 10.1056/NEJMoa054022 16807415

[38] Duntas LH Doumas C. The ‘rings of fire’ and thyroid cancer. Hormones (2009) 8(4):249–53. 10.14310/horm.2002.1242 20045797

[39] Truong T Baron-Dubourdieu D Rougier Y Guénel P. Role of dietary iodine and cruciferous vegetables in thyroid cancer: a countrywide case–control study in New Caledonia. Cancer Cause Control (2010) 21(8):1183–92. 10.1007/s10552-010-9545-2 PMC3496161 20361352

[40] Rong S Gao Y Yang Y Shao H Okekunle AP Lv C. Nitric oxide is involved in the hypothyroidism with significant morphology changes in female Wistar rats induced by chronic exposure to high water iodine from potassium iodate. Chemosphere (2018) 206:320–9. 10.1016/j.chemosphere.2018.05.015 29754056

[41] Hu X Moscinski L. Cdc2: a monopotent or pluripotent CDK? Cell Proliferation (2011) 44(3):205–11. 10.1111/j.1365-2184.2011.00753.x PMC6496858 21535261

[42] Cozzi M Giorgi F Marcelli E Pentimalli F Forte IM Schenone S. Antitumor activity of new pyrazolo[3,4-d]pyrimidine SRC kinase inhibitors in Burkitt lymphoma cell lines and its enhancement by WEE1 inhibition. Cell Cycle (Georgetown Tex) (2012) 11(5):1029–39. 10.4161/cc.11.5.19519 22333592

[43] Vara JÁF Casado E de Castro J Cejas P Belda-Iniesta C González-Barón M. PI3K/Akt signalling pathway and cancer. Cancer Treat Rev (2004) 30(2):193–204. 10.1016/j.ctrv.2003.07.007 15023437

[44] Martini M De Santis M Braccini L Gulluni F Hirsch E. PI3K/AKT signaling pathway and cancer: an updated review. Ann Med (2014) 46(6):372–83. 10.3109/07853890.2014.912836 24897931

