# Supplementary figures and images for: High Iodine Induces the Proliferation of Papillary and Anaplastic Thyroid Cancer Cells via AKT/Wee1/CDK1 Axis
Source: Front Oncol. 2021 Mar 16;11:622085. doi: 10.3389/fonc.2021.622085 (PMC8008130; doi:10.3389/fonc.2021.622085)

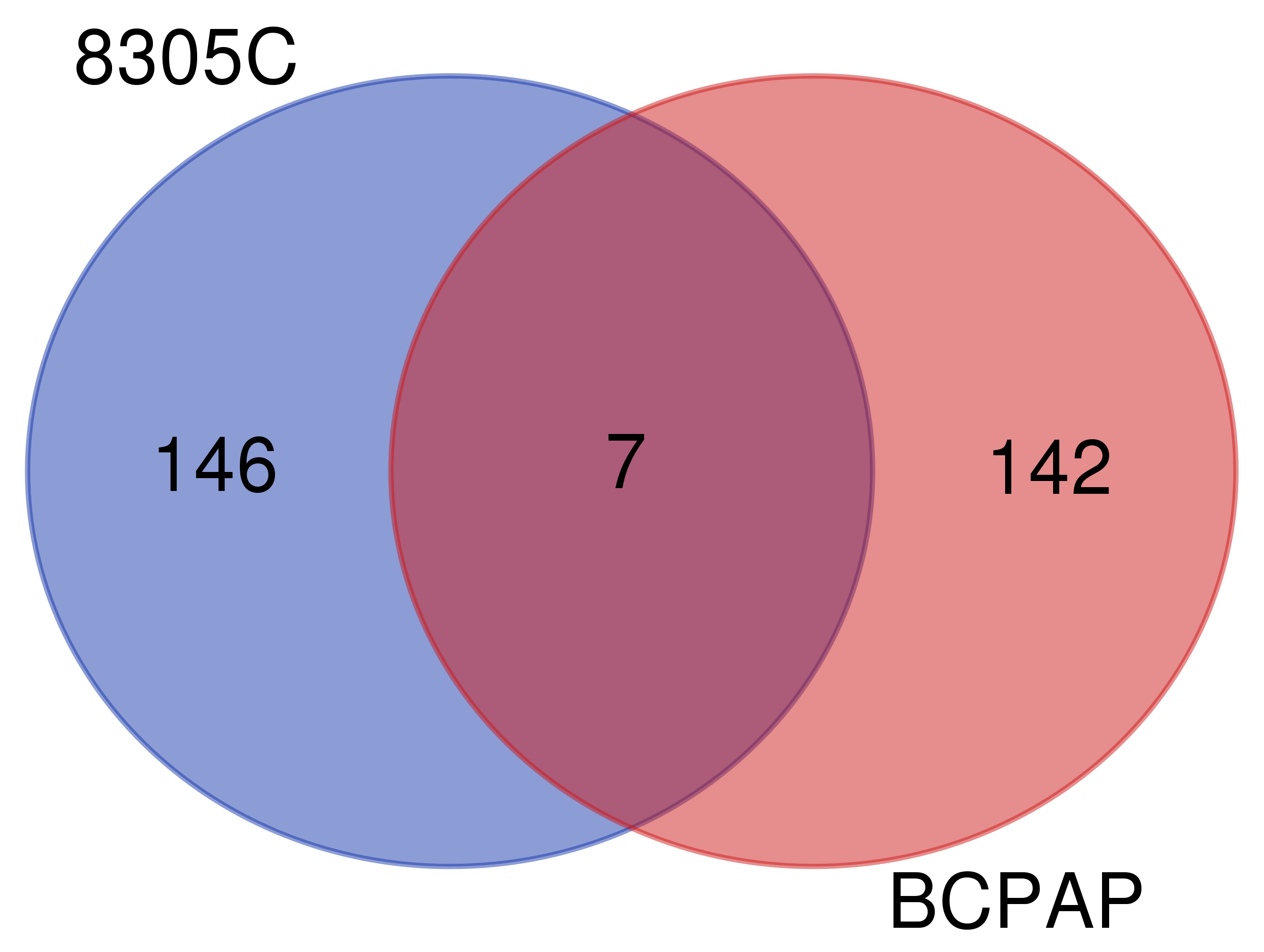

Supplement: Supplementary Figure 1 — The Venn calculation result obtained genes that involved in the proliferation of BCPAP and 8305C cells induced by high iodine. The 2 datasets showed an overlap of 295 genes. [file Image_1.png]
