# Supplementary material for: High Iodine Induces the Proliferation of Papillary and Anaplastic Thyroid Cancer Cells via AKT/Wee1/CDK1 Axis
Source: Front Oncol. 2021 Mar 16;11:622085. doi: 10.3389/fonc.2021.622085 (PMC8008130; doi:10.3389/fonc.2021.622085)
Supplement: Supplementary file 8 [file Table_1.doc]

**Table S1** Primer sequences for specific genes

| Gene | Primer pair sequences (5'–3') |
| --- | --- |
| AKT1 | F:GCAGGATGTGGACCAACGTGAG |
|  | R:GCAGGCAGCGGATGATGAAGG |
| Wee1 | F:ATGTGCTGCTGGTGCTGAACC |
|  | R:ACCAGTGCCATTGCTGAAGGTC |
| CDK1 | F:AGGAAGGGGTTCCTAGTACTGC |
|  | R:CCATGTACTGACCAGGAGGGA |
| β-actin | F:CATGTACGTTGCTATCCAGGC |
|  | R:CTCCTTAATGTCACGCACGAT |
